# Supplementary material for: Content-rich biological network constructed by mining PubMed abstracts
Source: BMC Bioinformatics. 2004 Oct 8;5:147. doi: 10.1186/1471-2105-5-147 (PMC528731; doi:10.1186/1471-2105-5-147)
Supplement: Additional File 2 — The original results of the above study (non-essential files are deleted to keep the file size under the limit set by BMC bioinformatics). [file 1471-2105-5-147-S2.bz2 › chilibotAdditionalFile2/dip05/213ID7790364E719/html/ITGA3_CD9.html]

 


 **ITGA3** and **CD9** 
  
Found 15 abstracts in PubMed, retrieved 05.  
 

 What does Google say? 
 PDF only 
| .edu only 

---

**Interactive relationship** (e.g. stimulation, inhibition, etc)

**Non-interactive relationship** (e.g. studied together, co-existance, homology, etc.)

- colon cancer progresses following collapse of the complex formed by integrin alpha3  [ **ITGA3** ]  with MRP 1  [ **CD9** ]   **CD9**  and KAI1 CD82.  Ref: 11802216 Int J Cancer, 2002
- A correlation was found between integrin alpha3  [ **ITGA3** ]  with MRP 1  [ **CD9** ]   **CD9**  and KAI1 CD82 for stage I tumors.  Ref: 11802216 Int J Cancer, 2002
- Immunoprecipitation followed by Western blot analysis showed association between  **CD9**  and integrin alpha3  [ **ITGA3** ]  in endometrium.  Ref: 12021064 Biol Reprod, 2002
- Data obtained are presented in concurrence with Clusters of Thymic Epithelial Staining CTES classification for various monoclonal antibodies recognizing CD antigens CD1, CD1a, CD6,  **CD9** , CD14, CD16, CD29, CD30, CD32, CD44, CD45RB, CD47, CD48, CD49a, CD49b, CD49c  [ **ITGA3** ] , CD49d, CD49e, CD49f, CD51, CD53, CD54, CD56, CD57, CD63, CD85, CD95, CD98, CD102, CD103, CD106, CD109, CD146, CD147, CD148, CD151, CD152, CD158a, CD158b, CD164, CD165, CD166 and for monoclonal antibodies 1B1 G7, A4, BD46, BLTZ, HP1C5, IND.64, M72, WU947 whose specifities are not yet defined.  Ref: 12725440 Ann Anat, 2003
- Furthermore, we investigated the correlation of integrin alpha3  [ **ITGA3** ]  with MRP 1  [ **CD9** ]   **CD9**  and KAI1 CD82.  Ref: 11802216 Int J Cancer, 2002
- Integrin alpha3  [ **ITGA3** ]  expression as a prognostic factor in colon cancer association with MRP 1  [ **CD9** ]   **CD9**  and KAI1 CD82.  Ref: 11802216 Int J Cancer, 2002
